# Case report: Fecal microbiota transplantation in refractory ankylosing spondylitis

**DOI:** 10.3389/fimmu.2023.1093233

**Published:** 2023-02-23

**Authors:** Lei Wang, Zhimin Wei, Fei Pan, Chuan Song, Lihua Peng, Yunsheng Yang, Feng Huang

**Affiliations:** ^1^ Department of Rheumatology and Immunology, The First Medical Center, Chinese PLA General Hospital, Beijing, China; ^2^ Health Service Department of the Guard Bureau of the General Office of the Central Committee of the Communist Party of China, Beijing, China; ^3^ Microbiota Division, Department of Gastroenterology and Hepatology, The First Medical Center, Chinese PLA General Hospital, Beijing, China

**Keywords:** ankylosing spondylitis, ulcerative colitis, fecal microbiota transplantation, gut microbiota, case report

## Abstract

Ankylosing spondylitis (AS) is the prototype of a group of systemic inflammatory diseases referred to as spondyloarthritis. Comorbid inflammatory bowel disease and changed gut microbiota in AS have attracted attention to the influence of gut–joint axis and encouraged treating AS by targeting gut microbiota. Here we first reported a patient with refractory AS and comorbid ulcerative colitis (UC) who underwent three fecal microbiota transplantations (FMTs). Inadequate response to conventional treatments including tumor necrosis factor inhibitors impelled FMT as alternative therapy. Notable improvements in AS and UC accompanied with changed fecal microbiota were recorded at 1 week post-FMT1. Further recovery was found after the other two FMTs, and a roughly stable status was maintained in the follow-up period. More studies are needed to validate the effectiveness of FMT in AS and its mechanisms.

## Introduction

Ankylosing spondylitis (AS) is the prototype of a broader class of systemic inflammatory diseases referred to as spondyloarthritis (SpA). Evidences of gut comorbidities, including inflammatory bowel disease (IBD) and functional bowel disease (FBD) (1, 2), and changed gut microbiota in AS prompt the important role of the gut–joint axis in the pathogenesis of AS (3). It was supposed that the effects of gut microbiota on AS were mediated through multiple ways including gut inflammation, expansion of mucosal Th17, migration of lymphocytes, and molecular mimicry (4–7). Explorations on an AS model had shown that gut microbiota and its metabolites, such as short-chain fatty acids (SCFAs), indole-3-acetate, and lipopolysaccharide, could influence the inflammatory status (8–11). However, there is still a lack of direct evidence showing the efficacy of therapies targeting gut microbiota on patients with AS. Here we describe a patient with AS and ulcerative colitis (UC) who underwent fecal microbiota transplantation (FMT).

## Case report

A 24-year-old male patient with a medical history of AS and UC visited our hospital. The patient, who had low back and hip pain for 6 months, was given a diagnosis of AS 8 years ago according to the 1984 modified New York criteria (12). Human leucocyte antigen B-27 (HLA-B27) was positive. His mother was diagnosed with UC many years ago, and his father had ever presented low back pain in his early 20s. Treated with loxoprofen, leflunomide, and Chinese herbal medicine, the symptoms got controlled. Due to recurrent episodes of low back pain and an elevated level of C-reactive protein (CRP) 7 years ago, etanercept was used to control the flare-up of AS.

Then, 3 years ago, with a presentation of continuous diarrhea and abdominal pain, the patient underwent a colonoscopy and was diagnosed with UC, the symptoms of which were controlled after the use of mesalazine.

Furthermore, the interruption of drugs for nearly 1 month flared the inactive disease1 year ago. Subsequently, adalimumab at 40 mg subcutaneously biweekly or etanercept at 50 mg subcutaneously every week, combined with acemetacin at 180 mg/day and mesalazine at 4 g/day, was used for over 3 months, but it failed to achieve an adequate response. Based on our previous successful FMT experience with UC (13), this patient was treated with three FMTs, which was proceeded 4 weeks after the last subcutaneous injection of etanercept to avoid infection risk.

Stools for FMTs were obtained from a 26-year-old healthy man (FMT1 and FMT2) and a 36-year-old healthy woman (FMT3), which had been screened through procedures including a preliminary questionnaire, physical examination, laboratory examinations, *etc.* (13). Briefly, a preliminary questionnaire including queries on medical history and lifestyle habits was used to exclude any exposure to infectious agents or risky behaviors, and laboratory examinations, including serology screening tests for HIV, hepatitis A, B, C, and E, syphilis, Epstein–Barr virus, cytomegalovirus, and rotavirus, and stool culturing for enteric pathogens including *Escherichia coli* O157, *Salmonella* spp., *Shigella* spp., *Campylobacter* spp., *Staphylococcus aureus*, *Yersinia*, *Vibrio parahaemolyticus*, *Vibrio cholerae*, *Candida albicans*, *Clostridium difficile toxin* A/B, ova, and parasites was performed to avoid a potential infection risk through FMT. On the day of each FMT, fresh stool (100 ± 20 g) was diluted with 500 ml normal saline, and the final volume of the fecal suspension was 400 ml after the procedures of suspension and filtration subsequently. In total, 100 ml of fecal suspension was infused into the jejunum by gastroscopy, and 300 ml was infused from the terminal ileum to the rectum by colonoscopy.

**Figure 1:**
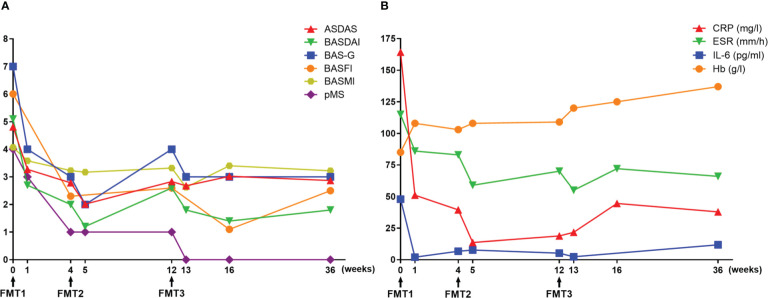
Therapeutic evaluation of fecal microbiota transplantation. **(A)** Clinical assessments of ankylosing spondylitis (AS) and ulcerative colitis (UC). **(B)** Laboratory findings. ASDAS, AS Disease Activity Score; BASDAI, Bath AS Disease Activity Index; BAS-G, Bath AS Patient Global Score; BASFI, Bath AS Functional Index; BASMI, Bath AS Metrology Index; pMS, partial Mayo score; CRP, C-reactive protein; ESR, erythrocyte sedimentation rate; IL-6, interleukin-6; Hb, hemoglobin.

**Figure 2:**
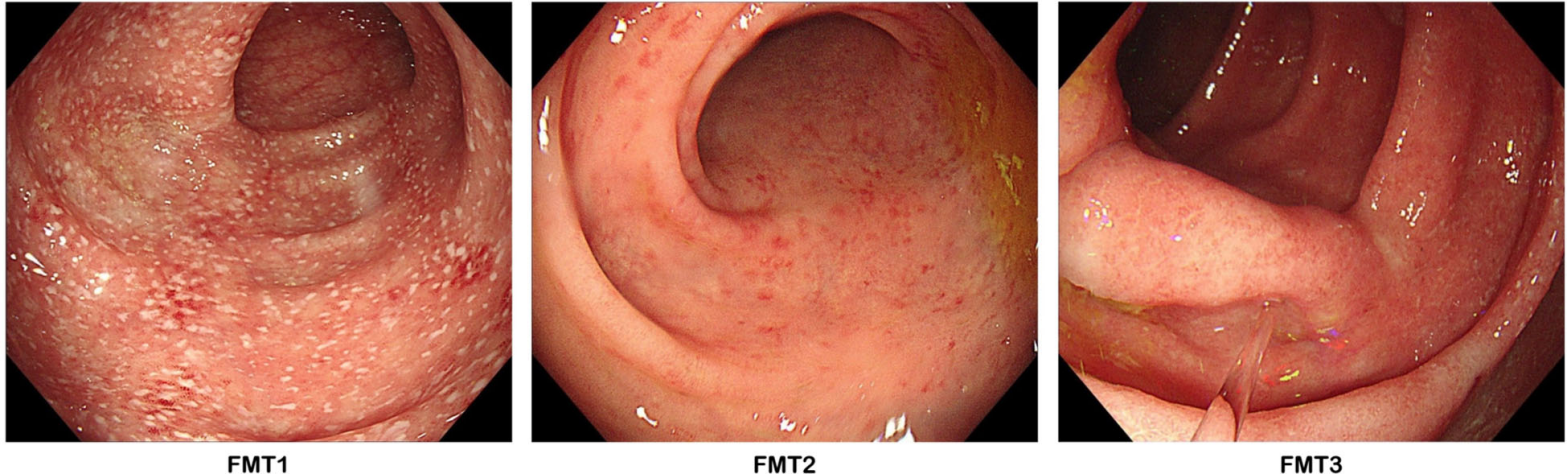
Colonoscopy photographs. FMT1 showing mucosal erythema, friability, erosions with white fur-like substance, and an obscure vascular pattern (Mayo endoscopic score = 2). FMT2 showing a milder degree of the above-mentioned characteristics (Mayo endoscopic score = 1). FMT3 showing an almost normal colonic mucosal appearance with faint inflammatory evidence (Mayo endoscopic score = 1).

At baseline, the assessments of AS showed a high disease activity, of which the AS Disease Activity Score based on C-reactive protein (ASDAS-CRP) and Bath Ankylosing Spondylitis Disease Activity Index (BASDAI) was 4.81 and 5.1, respectively (**Figure 1A**). Severe back pain with a limitation of axial mobility led the patient to be wheelchair-ridden. Besides this, a moderate disease activity of UC was exhibited (Mayo score = 6), in which bloody diarrhea occurred accompanied with a low level of hemoglobin (85 g/L). At 3 days after FMT1, the patient reported relief from low back pain. However, the right frontotemporal skin broke out in a rash, which faded 2 days later after using cetirizine hydrochloride. At 1 week after FMT1, dramatic improvements in AS as well as a reduction of bowel movements and abdominal pain were recorded. CRP decreased from a baseline of 164.3 to 51.15 mg/L. The erythrocyte sedimentation rate from baseline was 115 to 86 mm/h, and interleukin (IL)-6 from baseline was 47.95 to 2 pg/ml. Hemoglobin rose from a baseline value of 85 to 108 g/L (**Figure 1B**). Although the use of acemetacin was subsequently reduced to 90 mg/day, further improvements were noted at 4 weeks after FMT1 and at 1 week after FMT2 (week 5). Besides these, colonoscopy at FMT2 revealed slighter colitis (Mayo endoscopic sub-score from baseline 2 to 1) (**Figure 2**). The favorable recovery from AS and UC enabled the patient to restart his postgraduate study since 2 weeks before FMT3 (week 10), but staying up late led to a slight revival in AS. The symptoms of AS got controlled again after FMT3. During the follow-up period (week 16 and week 36), the AS disease status was maintained (**Figure 1**).

**Figure 3:**
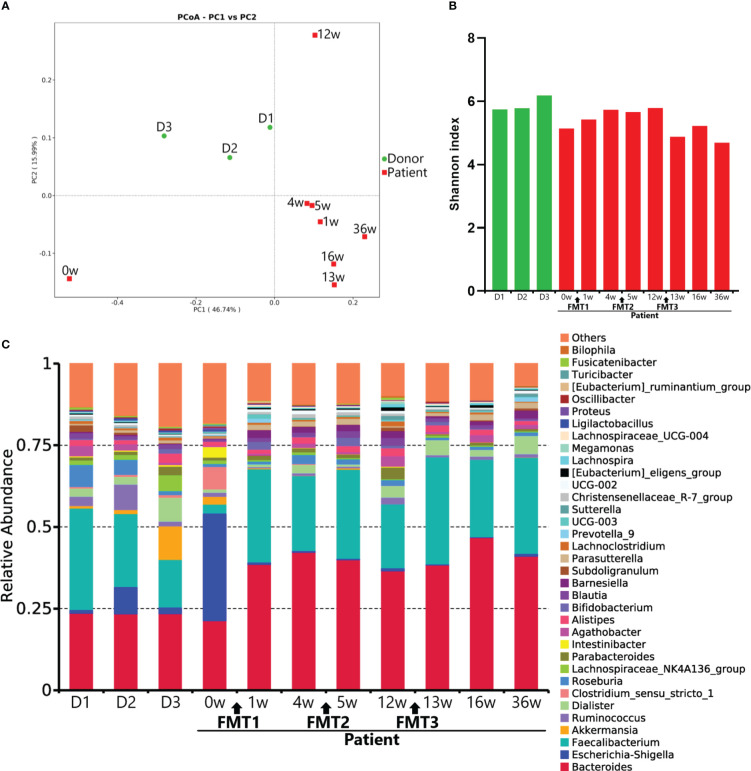
Microbial profiles. **(A)** Principal coordinate analysis of the taxonomy profile. **(B)** Shannon index of the gut microbiota. **(C)** Taxonomy at the genus level. D1–D3 represent the microbial profiles of donors for FMT1–3.

16S ribosomal RNA gene V4 region sequencing was performed on fecal DNA isolated from stool samples collected from the patient at serial evaluation points and from donors of FMTs for microbiota analysis. The results of the principal coordinate analysis demonstrated that the microbial profile of the patient improved toward those of donors following FMTs and remained roughly stable (**Figure 3A**). The Shannon index of the patient’s fecal microbiota was increased after FMT1 and decreased after FMT3 (**Figure 3B**). Marked changes at the genus level, such as decreased relative abundance of *Escherichia-Shigella* and *Intestinibacter* and increased relative abundance of *Faecalibacterium* and *Parasutterella*, were detected after FMT (**Figure 3C**). Moreover, a microbial disturbance at 8 weeks post-FMT2 (week 12), which might have been influenced by external factors such as staying up late, was also observed (**Figure 3**).

## Discussion

To our knowledge, this is the first report on treating refractory AS with FMT. For the treatment of inflammatory arthritis other than AS, there had been cases reporting the efficacy of FMT in psoriatic arthritis (PsA) and rheumatoid arthritis (RA) (14, 15). Incidental improvements of back pain and morning stiffness after FMT were also reported by one UC patient without sacroiliitis (16), whereas a positive efficacy of FMT on PsA was not found in a randomized placebo-controlled trial (17).

Conventionally, the donor is of vital importance to the safety and efficacy of FMT. For the present case, although the donors had undergone adequate examinations to avoid potential infectious risk through FMT (13), a cutaneous hypersensitivity reaction appeared to the patient after FMT1. In retrospect, a similar skin rash had previously occurred once to the patient after the consumption of mango, which, coincidentally, was also consumed by the donor 2 days before the stool donation. Hence, it was speculated that FMT might have transferred some components causing the allergic reaction in the patient but not in the donor, indicating that a patient’s factors should be considered in the dietary management of the donor to avoid relative risk. In addition, replacement of a donor should not be neglected as among the possible reasons why no more significant improvement was found after FMT3.

Any procedure of FMT, from defecation of the donor to infusion of fecal suspension into the recipient, might also influence the outcome. In view of previous studies, we chose procedures which included fresh stools rather than frozen stools (18), three doses rather than only one (19), and multiple routes (*via* colonoscopy and gastroscopy) rather than a single one (20) to protect the microbial efficacy from disturbance in the process as far as possible.

The microbial changes after FMT in the present case, including the increased amount of *Parasutterella* (21–24) and the decreased amounts of *Escherichia–Shigella* (23, 25) and *Intestinibacter* (21, 26), were in accordance with the results of studies comparing the gut microbiota of cases with AS *versus* healthy controls. However, the amount of the genus *Faecalibacterium*, which was previously reported to be enriched in patients with AS compared with controls (25, 27), was increased, accompanied with a decreased disease activity after FMT. Studies on *Faecalibacterium* had reported its anti-inflammatory characteristics such as maintaining Th17/Treg balance, blocking NF-kappaB activation, and increasing IL-10 production (28, 29). In addition, the deficiency of *Faecalibacterium* was also observed in patients with early RA (30). Hence, *Faecalibacterium* might be protective to AS, and the paradoxical results were probably attributed to the medication use of patients in previous case–control studies. Except for taxa related to the disease activity of the patient, other taxa such as *Streptococcus* (21, 25, 26), *Dialister* (23, 26, 31), and *Clostridium bolteae* (22, 32) might also have potential impacts on AS, which need explorations to testify the causal relationship between specific bacteria and the progression of AS.

The slight relapse of AS at 4 weeks post-FMT2, accompanied with a deviation of the fecal microbial profile, elicited a further question about the achievement of durable remission. External factors could influence the gut microbiota. Poor lifestyle such as staying up late could influence normal diurnal rhythmicity exhibited in the gut microbiota and its metabolites to cause fatigue (33), and then it might lead to a flare of AS (34). Diet could influence both the disease activity of AS and gut microbiota. High dietary fiber intake might be helpful to maintain the efficacy of FMT through promoting beneficial bacteria and increasing the production of SCFAs (35). Moreover, a successful combination of FMT and a multidimensional sulfide-reducing diet on one patient with UC supported a defined diet that might also be conducive to prolong the efficacy of FMT (36). Prebiotics was also reported to improve the anti-inflammatory effect of FMT (37). Drugs for AS, including non-steroidal anti-inflammatory drug and tumor necrosis factor inhibitor, could change the gut microbiota (23, 38, 39); however, whether FMT and drugs could mutually strengthen the efficacy on AS remains unknown and is worthy of exploration.

Collectively, the present case shows a notable efficacy of FMT in refractory AS with comorbid UC, which deserves cohort studies with large samples. Further exploration on strains or microbial metabolites related to AS is beneficial to the advancement of FMT in donor selection, durable remission, and even replacement of fecal suspension by certain elements.

## Patient’s perspective

The alternative therapy, FMTs, alleviated me from refractory AS and UC which had afflicted me for almost 1 year. Now, I am feeling fine in the mass and could continue my postgraduate study, even though low back pain sometimes disturbs me accompanied with laborious work and cold weather. I hope that my case inspires more doctors to advance therapies on AS.

## Data availability statement

The datasets presented in this study can be found in online repositories. The name of the repository and accession number can be found below: PRJNA900383 (SRA).

## Ethics statement

The studies involving human participants were reviewed and approved by the Ethics Committee of Chinese PLA General Hospital. The patients/participants provided their written informed consent to participate in this study. Written informed consent was obtained for the publication of this case report.

## Author contributions

FH, YY, and ZW contributed to the conception and design of the study. LW and CS contributed to data collection. FP prepared the fecal suspensions. YY and LP performed fecal microbiota transplantations. LW and ZW drafted the article. FH critically revised the manuscript. All authors contributed to the article and approved the submitted version.

## References

[1] Stolwijk C van Tubergen A Castillo-Ortiz JD Boonen A. Prevalence of extra-articular manifestations in patients with ankylosing spondylitis: a systematic review and meta-analysis. Ann Rheum Dis (2015) 74(1):65–73. doi: 10.1136/annrheumdis-2013-203582 23999006

[2] Wang L Song C Wang Y Hu L Liu X Zhang J. Symptoms compatible with Rome IV functional bowel disorder in patients with ankylosing spondylitis. Mod Rheumatol (2022) 21:roac064. doi: 10.1093/mr/roac064 35727178

[3] Wang L Wang Y Zhang P Song C Pan F Li G. Gut microbiota changes in patients with spondyloarthritis: A systematic review. Semin Arthritis Rheum (2022) 52:151925. doi: 10.1016/j.semarthrit.2021.11.002 34844732

[4] Hsieh WC Svensson MN Zoccheddu M Tremblay ML Sakaguchi S Stanford SM. PTPN2 links colonic and joint inflammation in experimental autoimmune arthritis. JCI Insight (2020) 5(20):e141868. doi: 10.1172/jci.insight.141868 33055428 PMC7605542

[5] Asquith MJ Stauffer P Davin S Mitchell C Lin P Rosenbaum JT. Perturbed mucosal immunity and dysbiosis accompany clinical disease in a rat model of spondyloarthritis. Arthritis Rheumatol (2016) 68(9):2151–62. doi: 10.1002/art.39681 PMC5542398 26992013

[6] Ansalone C Utriainen L Milling S Goodyear CS. Role of gut inflammation in altering the monocyte compartment and its osteoclastogenic potential in HLA-B27-Transgenic rats. Arthritis Rheumatol (2017) 69(9):1807–15. doi: 10.1002/art.40154 28511292

[7] Zhang L Hu Y Xu Y Li P Ma H Li X. The correlation between intestinal dysbiosis and the development of ankylosing spondylitis. Microb Pathog (2019) 132:188–92. doi: 10.1016/j.micpath.2019.04.038 31039390

[8] Ciccia F Guggino G Zeng M Thomas R Ranganathan V Rahman A. Proinflammatory CX3CR1+CD59+Tumor necrosis factor-like molecule 1A+Interleukin-23+ monocytes are expanded in patients with ankylosing spondylitis and modulate innate lymphoid cell 3 immune functions. Arthritis Rheumatol (2018) 70(12):2003–13. doi: 10.1002/art.40582 29869839

[9] Gill T Brooks SR Rosenbaum JT Asquith M Colbert RA. Novel inter-omic analysis reveals relationships between diverse gut microbiota and host immune dysregulation in HLA-B27-Induced experimental spondyloarthritis. Arthritis Rheumatol (2019) 71(11):1849–57. doi: 10.1002/art.41018 PMC7603391 31216122

[10] Berlinberg AJ Regner EH Stahly A Brar A Reisz JA Gerich ME. Multi 'Omics analysis of intestinal tissue in ankylosing spondylitis identifies alterations in the tryptophan metabolism pathway. Front Immunol (2021) 12:587119. doi: 10.3389/fimmu.2021.587119 33746944 PMC7966505

[11] Capkova J Hrncir T Kubatova A Tlaskalova-Hogenova H. Lipopolysaccharide treatment suppresses spontaneously developing ankylosing enthesopathy in B10.BR male mice: the potential role of interleukin-10. BMC Musculoskelet Disord (2012) 13:110. doi: 10.1186/1471-2474-13-110 22721554 PMC3493365

[12] van der Linden S Valkenburg HA Cats A. Evaluation of diagnostic criteria for ankylosing spondylitis. a proposal for modification of the new York criteria. Arthritis Rheum (1984) 27(4):361–8. doi: 10.1002/art.1780270401 6231933

[13] Ren R Gao X Shi Y Li J Peng L Sun G. Long-term efficacy of low-intensity single donor fecal microbiota transplantation in ulcerative colitis and outcome-specific gut bacteria. Front Microbiol (2021) 12:742255. doi: 10.3389/fmicb.2021.742255 34867859 PMC8635752

[14] Selvanderan SP Goldblatt F Nguyen NQ Costello SP. Faecal microbiota transplantation for clostridium difficile infection resulting in a decrease in psoriatic arthritis disease activity. Clin Exp Rheumatol (2019) 37(3):514–5. 30943129

[15] Zeng J Peng L Zheng W Huang F Zhang N Wu D. Fecal microbiota transplantation for rheumatoid arthritis: A case report. Clin Case Rep (2020) 9(2):906–9. doi: 10.1002/ccr3.3677 PMC7869316 33598269

[16] Mahajan R Midha V Singh A Mehta V Gupta Y Kaur K. Incidental benefits after fecal microbiota transplant for ulcerative colitis. Intest Res (2020) 18(3):337–40. doi: 10.5217/ir.2019.00108 PMC7385574 32306706

[17] Kragsnaes MS Kjeldsen J Horn HC Munk HL Pedersen JK Just SA. Safety and efficacy of faecal microbiota transplantation for active peripheral psoriatic arthritis: an exploratory randomised placebo-controlled trial. Ann Rheum Dis (2021) 80(9):1158–67. doi: 10.1136/annrheumdis-2020-219511 33926922

[18] Cheng F Huang Z Wei W Li Z. Fecal microbiota transplantation for crohn's disease: a systematic review and meta-analysis. Tech Coloproctol (2021) 25(5):495–504. doi: 10.1007/s10151-020-02395-3 33759066

[19] Singh R de Groot PF Geerlings SE Hodiamont CJ Belzer C Berge IJMT. Fecal microbiota transplantation against intestinal colonization by extended spectrum beta-lactamase producing enterobacteriaceae: a proof of principle study. BMC Res Notes (2018) 11(1):190. doi: 10.1186/s13104-018-3293-x 29566738 PMC5863815

[20] Saïdani N Lagier JC Cassir N Million M Baron S Dubourg G. Faecal microbiota transplantation shortens the colonisation period and allows re-entry of patients carrying carbapenamase-producing bacteria into medical care facilities. Int J Antimicrob Agents (2019) 53(4):355–61. doi: 10.1016/j.ijantimicag.2018.11.014 30472293

[21] Zhang L Han R Zhang X Fang G Chen J Li J. Fecal microbiota in patients with ankylosing spondylitis: Correlation with dietary factors and disease activity. Clin Chim Acta (2019) 497:189–96. doi: 10.1016/j.cca.2019.07.038 31377126

[22] Yin J Sternes PR Wang M Song J Morrison M Li T. Shotgun metagenomics reveals an enrichment of potentially cross-reactive bacterial epitopes in ankylosing spondylitis patients, as well as the effects of TNFi therapy upon microbiome composition. Ann Rheum Dis (2020) 79(1):132–40. doi: 10.1136/annrheumdis-2019-215763 31662318

[23] Zhang F Ma C Zhang B. Dynamic variations in gut microbiota in ankylosing spondylitis patients treated with anti-TNF-α for six months. Ann Clin Lab Sci (2020) 50(1):99–106. 32161018

[24] Liu G Hao Y Yang Q Deng S. The association of fecal microbiota in ankylosing spondylitis cases with c-reactive protein and erythrocyte sedimentation rate. Mediators Inflamm (2020) 2020:8884324. doi: 10.1155/2020/8884324 33204218 PMC7666627

[25] Li M Dai B Tang Y Lei L Li N Liu C. Altered bacterial-fungal interkingdom networks in the guts of ankylosing spondylitis patients. mSystems (2019) 4(2):e00176–18. doi: 10.1128/mSystems.00176-18 PMC6435815 30944880

[26] Chen Z Qi J Wei Q Zheng X Wu X Li X. Variations in gut microbial profiles in ankylosing spondylitis: disease phenotype-related dysbiosis. Ann Transl Med (2019) 7(20):571. doi: 10.21037/atm.2019.09.41 31807552 PMC6861740

[27] Wen C Zheng Z Shao T Liu L Xie Z Le Chatelier E. Quantitative metagenomics reveals unique gut microbiome biomarkers in ankylosing spondylitis. Genome Biol (2017) 18(1):142. doi: 10.1186/s13059-017-1271-6 28750650 PMC5530561

[28] Zhou L Zhang M Wang Y Dorfman RG Liu H Yu T. Faecalibacterium prausnitzii produces butyrate to maintain Th17/Treg balance and to ameliorate colorectal colitis by inhibiting histone deacetylase 1. Inflamm Bowel Dis (2018) 24(9):1926–40. doi: 10.1093/ibd/izy182 29796620

[29] Delgado S Sánchez B Margolles A Ruas-Madiedo P Ruiz L. Molecules produced by probiotics and intestinal microorganisms with immunomodulatory activity. Nutrients (2020) 12(2):391. doi: 10.3390/nu12020391 32024101 PMC7071221

[30] Chu XJ Cao NW Zhou HY Meng X Guo B Zhang HY. The oral and gut microbiome in rheumatoid arthritis patients: a systematic review. Rheumatol (Oxford) (2021) 60(3):1054–66. doi: 10.1093/rheumatology/keaa835 33450018

[31] Costello ME Ciccia F Willner D Warrington N Robinson PC Gardiner B. Brief report: Intestinal dysbiosis in ankylosing spondylitis. Arthritis Rheumatol (2015) 67(3):686–91. doi: 10.1002/art.38967 25417597

[32] Huang R Li F Zhou Y Zeng Z He X Fang L. Metagenome-wide association study of the alterations in the intestinal microbiome composition of ankylosing spondylitis patients and the effect of traditional and herbal treatment. J Med Microbiol (2020) 69(6):797–805. doi: 10.1099/jmm.0.001107 31778109 PMC7451032

[33] Matenchuk BA Mandhane PJ Kozyrskyj AL. Sleep, circadian rhythm, and gut microbiota. Sleep Med Rev (2020) 53:101340. doi: 10.1016/j.smrv.2020.101340 32668369

[34] Zhou W Guo J He M Li J Chen Y Liu J. Fatigue and contributing factors in Chinese patients with ankylosing spondylitis. Clin Rheumatol (2020) 39(8):2337–44. doi: 10.1007/s10067-020-04976-x 32133565

[35] Vergne-Salle P Salle L Fressinaud-Marie AC Descamps-Deplas A Montestruc F Bonnet C. Diet and disease activity in patients with axial spondyloarthritis: SpondyloArthritis and NUTrition study (SANUT). Nutrients (2022) 14(22):4730. doi: 10.3390/nu14224730 36432416 PMC9695957

[36] Bryant RV Day AS McGrath KC Telfer K Yao CK Costello SP. Fecal microbiota transplantation augmented by a sulfide-reducing diet for refractory ulcerative colitis: A case report with functional metagenomic analysis. JGH Open (2021) 5(9):1099–102. doi: 10.1002/jgh3.12623 PMC8454482 34584982

[37] Xi M Li J Hao G An X Song Y Wei H. Stachyose increases intestinal barrier through akkermansia muciniphila and reduces gut inflammation in germ-free mice after human fecal transplantation. Food Res Int (2020) 137:109288. doi: 10.1016/j.foodres.2020.109288 33233042

[38] Maseda D Ricciotti E. NSAID-gut microbiota interactions. Front Pharmacol (2020) 11:1153. doi: 10.3389/fphar.2020.01153 32848762 PMC7426480

[39] Chen Z Zheng X Wu X Wu J Li X Wei Q. Adalimumab therapy restores the gut microbiota in patients with ankylosing spondylitis. Front Immunol (2021) 12:700570. doi: 10.3389/fimmu.2021.700570 34539629 PMC8441001

